# Transcriptome-Wide Identification of Reference Genes for Expression Analysis of Soybean Responses to Drought Stress along the Day

**DOI:** 10.1371/journal.pone.0139051

**Published:** 2015-09-25

**Authors:** Juliana Marcolino-Gomes, Fabiana Aparecida Rodrigues, Renata Fuganti-Pagliarini, Thiago Jonas Nakayama, Rafaela Ribeiro Reis, Jose Renato Bouças Farias, Frank G. Harmon, Hugo Bruno Correa Molinari, Mayla Daiane Correa Molinari, Alexandre Nepomuceno

**Affiliations:** 1 Embrapa Soybean, Brazilian Agricultural Research Corporation, Londrina, Paraná, Brazil; 2 Department of Biology, State University of Londrina, Londrina, Paraná, Brazil; 3 Department of Crop Science, Federal University of Viçosa, Viçosa, Minas Gerais, Brazil; 4 Plant Gene Expression Center, ARS/USDA, Albany, California, United States of America; 5 Department of Plant and Microbial Biology, University of California-Berkeley, Berkeley, California, United States of America; 6 Embrapa Agroenergy, Brazilian Agricultural Research Corporation, Brasília, Brazil; Institute for Sustainable Plant Protection, C.N.R., ITALY

## Abstract

The soybean transcriptome displays strong variation along the day in optimal growth conditions and also in response to adverse circumstances, like drought stress. However, no study conducted to date has presented suitable reference genes, with stable expression along the day, for relative gene expression quantification in combined studies on drought stress and diurnal oscillations. Recently, water deficit responses have been associated with circadian clock oscillations at the transcription level, revealing the existence of hitherto unknown processes and increasing the demand for studies on plant responses to drought stress and its oscillation during the day. We performed data mining from a transcriptome-wide background using microarrays and RNA-seq databases to select an unpublished set of candidate reference genes, specifically chosen for the normalization of gene expression in studies on soybean under both drought stress and diurnal oscillations. Experimental validation and stability analysis in soybean plants submitted to drought stress and sampled during a 24 h timecourse showed that four of these newer reference genes (*FYVE*, *NUDIX*, *Golgin-84* and *CYST*) indeed exhibited greater expression stability than the conventionally used housekeeping genes (*ELF1-β* and *β-actin*) under these conditions. We also demonstrated the effect of using reference candidate genes with different stability values to normalize the relative expression data from a drought-inducible soybean gene (*DREB5*) evaluated in different periods of the day.

## Introduction

As sessile organisms, plants must endure environmental changes during the day and across seasons. These environmental oscillations strongly affect light, temperature, nutrient and water availability, acting as a powerful selective pressure that have shaped adaptive mechanisms in plants during their evolutionary history. As a result, these organisms have developed a complex molecular network that confers adaptive advantages by coordinating their metabolism with predictable daily and seasonal changes, known as the circadian clock [1].

The circadian clock is composed of a core of interconnected transcriptional–translational feedback loops, which are entrained by signals such as light and temperature to adjust metabolism to the environment. In plants, the clock controls a number of physiological and developmental processes. For example, the expression of chlorophyll biosynthesis genes is regulated by the circadian clock to peak at the end of the night, which is an important mechanism to ensure photosynthesis in subsequent light periods of the day, whereas the products of photosynthesis modulate the rhythm [2]. The circadian clock also allows plants to coordinate flowering with favorable seasons to increase their fitness [1], as well as it controls the rate of starch degradation [3] and nitrogen assimilation and utilization pathways [4].

In addition to normal day/night variations, plants are subject to other environmental variations via biotic and abiotic stresses. Among the abiotic stresses, drought stands out as the factor with the greatest impact on yield of important crops worldwide, including soybean. Different mechanisms are employed by plants to protect themselves against water deficits, including changes in stomatal conductance [5], osmotic adjustment [6], the accumulation of osmoprotectant molecules [7], and the activity of antioxidant proteins [8]. Because the circadian clock is known to improve organism fitness according to environmental conditions, a significant number of studies addressing the relationships between water deficit stress and the circadian clock have been conducted, providing consistent evidence of this interaction [9–13].

The metabolic and physiological adjustments performed in response to drought stresses usually involve the reconfiguration of the transcriptome [12,13], and therefore the analysis of gene expression in response to water deficits during the day is an interesting strategy [10]. One of the most sensitive methods for the quantification of gene expression is the fluorescence-based quantitative real-time PCR (RT-qPCR), which is increasingly being used. The advantages of this technique include its practical simplicity combined with the possibility of measuring small amounts of RNA in a wide range of samples, rapidly and with high specificity.

Thus, RT-qPCR is an important tool that allows the relative quantification of transcript abundance and can therefore be used to evaluate gene expression responses to environmental changes, such as diurnal oscillations and abiotic stresses, including drought. However, because most of the quantitative RNA data obtained are not absolute, but relative, accurate quantification of gene expression relies on the use of appropriate reference genes. These genes should be stably expressed, showing a transcript abundance that is strongly correlated with the total mRNA present in the samples to allow the normalization of gene expression data [14]. Normalization is a key step in RT-qPCR analysis, as it reduces/eliminates variations due to variations in RNA extraction, reverse transcription yields or amplification efficiency, allowing comparisons of mRNA concentrations across different samples, playing a critical role in the accurate quantification of relative gene expression [14]. Although several genes have been indicated as good references, it is known that even housekeeping genes may exhibit altered expression in response to experimental treatments, sampling times and the life cycle [15–18].

In this context, a reference gene must be experimentally validated for specific tissues, genotypes and experimental designs. The soybean genes *TUA* (Glyma08g12140), *TUB* (Glyma03g27970), *ELF1-β* (Glyma13g04050), *β-actin* (Glyma15g05570) and *GAPDH* (Glyma06g01850) have been widely used as references in gene expression studies on drought responses [15,19]. On the other hand, isopentenyl diphosphate (*IPP2*), actin and ubiquitin are the most commonly used reference genes in studies investigating circadian/diurnal oscillations [20–25]. Thus, no study conducted to date has evaluated the expression stability of reference genes for the study of both water deficit stress and circadian oscillations in soybean. Hence, in this study, after evaluating gene expression in response to drought during the day, we present a novel set of reference genes suitable for the normalization of relative expression data from combined studies on water deficit and diurnal oscillations.

## Material and Methods

### Selection of reference genes using the RefGenes tool

To evaluate the stability of genes expressed in response to drought during the day, we used the RefGenes tool from the Genevestigator platform [26], available at [https://www.genevestigator.com/gv/plant.jsp]. The Genevestigator platform provides a database of normalized and well-annotated microarray experiments, allowing asses the transcriptome of several organisms; the RefGenes tool enables searching for genes with minimal expression variance across a chosen set of arrays at the Genevestigator platform. For the purposes of this study we performed analysis of gene expression variance in 59 microarray libraries from soybean subjected to drought, heat and distinct light periods. To select the candidate reference genes presenting range of expression levels detected by RT-qPCR, we uploaded a list of the Gene Models (“Glyma”) from reference genes commonly used for gene expression normalization in soybean, previously described by Hu and colleagues (2009) [19], presented in Table 1.

**Table 1:** Commonly used RT-qPCR reference genes from soybean, according to Hu and colleagues (2009).

Gene name	Gene Model	Description
***CYP***	Glyma12g02790	Cyclophilin
***TUB4***	Glyma03g27970	beta Tubulin
***SKIP16***	Glyma12g05510	Ask-Interacting Protein 16
***PEPKR1***	Glyma10g38460	Phosphoenolpyruvate Carboxylase-Related Kinase 1
***TIP41***	Glyma20g26690	TIP41-like family protein
***ELF1-β***	Glyma13g04050	elongation factor 1-B
***TUA***	Glyma08g12140	alpha tubulin
***β-actin***	Glyma15g05570	actin
***GAPDH***	Glyma06g01850	GAPDH

### Selection of reference genes using RNA-seq libraries

Under a second approach, we evaluated the expression stability of genes from 36 RNA-seq libraries. These libraries were synthesized from leaves of the drought-sensitive soybean genotype BR16, subjected to moderate drought stress on V2 developmental stage, sampled over a 24 h timecourse, with 4h intervals [10]. These data are deposited in the NCBI’s Gene Expression Omnibus [GEO; http://www.ncbi.nlm.nih.gov/geo/] repository and are accessible through GEO Series accession number GSE69469 (geospiza.com/Products/AnalysisEdition.shtml). To compare gene expression between different times and conditions, we log_2_-transformed the normalized reads per mapped million (RPM) value.

In this analysis, we selected genes that exhibited minimal expression variance across the libraries, presenting Coefficient of variation lower than 5%, with a range of expression similar to that of commonly used soybean reference genes[19], presented in Table 1.

### Primer design

Primers for the new candidate reference genes were designed based on soybean Gene Model sequences [http://www.phytozome.net/search.php?method=Org_Gmax] using the program Primer3 Plus [27], available at [http://www.bioinformatics.nl/cgi-bin/primer3plus/primer3plus.cgi]. The primer sequences were determined for the 3’ end of each gene whenever possible, and the amplicons spanned up to 150 base pairs (bp). The primer sequences were subjected to BLAST searches against the soybean genome [http://www.phytozome.net/search.php?method=Org_Gmax] to verify the specificity of each primer, as recommended by the Minimum Information for Publication of Quantitative Real-Time PCR Experiments guideline (MIQE) [14]. The primers for the commonly used reference genes *ELF1-β* and *β-actin* were selected from [28] and [29], respectively. The primers for the target gene (*GmDREB5*) were selected from [30]. Standard curves were produced from serial dilutions of a cDNA pool to estimate the efficiency of the PCR amplification with each pair of primers. Information on the primers may be visualized in Table 2.

**Table 2:** Information on reference and target genes.

Gene name	Gene Model	Sense primer	Primer sequence	Temperature melting (°C)	Amplicon size (bp)	Amplification Efficiency (%)	Primer Concentration (nM)
*NUDIX* ^*a*^	Glyma13g24060	F	TGAGTGTTAGAAGGGCTACTGG	58.5	108	88.02	200
* *		R	AACTTTGCCAACGGCATC	59.7			
*CYST* ^*a*^	Glyma01g40510	F	TTCTTGGATCGGGGAGAG	58.7	126	95.49	100
* *		R	GCTAGAAATGGCGAAAGAGG	59.1			
*FYVE* ^*a*^	Glyma13g17500	F	TTCTGTCTTCTGCAAGTGGTG	59.1	92	98.37	100
* *		R	GATCCCTCATCCATACATTTCAG	59.7			
*Golgin-84* ^*a*^	Glyma08g05790	F	TTGGACAAGGAGAGACTCCAC	59.3	121	98.57	100
* *		R	TGCGAGGCTACGAAAACTTC	60.5			
*NCL1* ^*a*^	Glyma11g38000	F	TCTTATCGGCATGGTTACGC	61.0	72	96.38	100
* *		R	ACATAACCAAACGCCAAAGC	60.0			
*RNA-poly Mitovirus* ^*a*^	Glyma08g41240	F	TCTCATCACCGATCCACTTG	59.6	114	98.72	100
* *		R	GCAAACTCTACAGCACCAGTTG	60.0			
*DNAJ* ^*a*^	Glyma10g44020	F	GATTGGGATGTTCTTCACCAG	59.4	136	90.50	200
* *		R	ATGACCAAGCCGATGGTTAG	60.0			
*ELF1-β* ^*b*^	Glyma13g04050	F	GTTGAAAAGCCAGGGGACA	58.0	118	99.16	60
* *		R	TCTTACCCCTTGAGCGTGG	58.0			
*β-actin* ^*b*^	Glyma15g05570	F	GAGCTATGAATTGCCTGATGG	58.0	118	97.92	60
* *		R	CGTITCATGAATTCCAGTAGC	55.0			
*DREB5* ^*c*^	Glyma12g33020	F	TTGCCTACTACTACTCCTATATTCATTTCC	58.0	86	97.80	100
* *		R	CCTTGAAATACACGGAGCCTTAG	58.0			

^a^ New reference candidate genes

^b^ Commonly used reference genes

^c^ Target gene

### Plant material and treatment application

Plant material was obtained from experiments performed as described by Marcolino-Gomes and colleagues [10]. Briefly, seeds from the soybean BR16 genotype, which exhibits drought-sensitive characteristics [31], were cultivated in peat pots (Jiffy) with Supersoil® (Scotts Miracle-Gro Company, Marysville, Ohio, USA) under optimal growth conditions in controlled growth chambers until reaching the V_2_ developmental stage [32], when water was withheld to induce a moderate water deficit. Control plants were maintained near field capacity for the unstressed treatment. The soil moisture was calculated by the gravimetric humidity (GH), which corresponds to the percentage of water in the soil in relation to the dry weight of the soil. The volume of irrigation was adjusted to 70% (GH) (near field capacity) for the unstressed treatment, 30% GH for the moderate stress treatment. The pots were weighed twice a day, and water was added to maintain the treatments at the desired GH values. The middle leaflet of the first trifoliate leaf was collected from plants in each treatment group at 4 h intervals from the time the lights came on (8:00 a.m. = Zeitgeiber Time (ZT) 0), during a 24 h timecourse (ZT0, ZT4, ZT8, ZT12, ZT16 and ZT20), and were immediately frozen in liquid nitrogen and stored at −80°C until further use. All of the experiments were conducted with three biological replicates, with each replicate consisting of two plants, whose tissues were collected together and pooled.

### From RNA extraction to cDNA synthesis

Each replicate tissue set was ground to a fine powder in liquid nitrogen, and total RNA was isolated using the TRIzol reagent (Invitrogen) according to the manufacturer’s instructions. The obtained RNA concentration and purity were measured using a spectrophotometer (NanoDrop, ND-1000), and contaminating DNA in the total RNA was removed using the Turbo DNA-free kit, according to the manufacturer’s instructions (Life Technologies, Grand Island, NY, USA). After DNAse treatment, the integrity of the molecules was analyzed on 1% agarose gels stained with ethidium bromide, and high-quality total RNA was used to synthesize cDNA strands (Superscript III First Strand Synthesis, Invitrogen/Life Technologies, Grand Island, NY, USA). The quality of the cDNA and contamination with genomic DNA were examined using a standard PCR assay with primers that spanned an intronic region of the *β-actin* soybean gene. High-quality cDNA was used to analyze the transcripts in each treatment.

### RT-qPCR analyses

Standard curves were produced from serial dilutions of a cDNA pool to estimate the efficiency of the PCR amplification with each pair of primers. RT-qPCR amplifications were performed in a 7300 RT-qPCR Thermocycler (Applied Biosystems/Life Technologies, Grand Island, NY, USA) with the following cycling parameters: 50°C for 2 min, 95°C for 10 min and 45 cycles at 95°C for 2 min, 60°C for 30 seconds and 72°C for 30 seconds. Each amplification reaction contained 2 μL of cDNA from serial dilutions, 60–200 nM each forward and reverse primer (Table 2), 500 nM ROX (passive reference), 6.5 μL of Platinum^®^ SYBR^®^ Green qPCR SuperMix (Invitrogen/ Grand Island, NY, USA), and ultrapure water to a final volume of 12.5 μL. Data were collected during the extension phase, and dissociation curves were generated by heating each reaction from 60 to 95°C and taking readings at one-degree intervals to verify the specificity of the primers. A control sample, obtained via performing RT-qPCR with no template, was also assayed to confirm that the samples were not contaminated. The primer concentrations were adjusted to achieve efficiency rates higher than 85%, as detailed in Table 2.

After carrying out the efficiency analysis, the expression levels of the candidate reference genes were analyzed separately at 6 time points (ZT0, ZT4, ZT8, ZT12, ZT16 and ZT20) in plants under control *vs*. drought stress condition in order to assess their expression stability along the day. The expression of the target gene (*GmDREB5*-like; Glyma12g33020) [30] was also measured under the experimental conditions described above. The reactions were performed in triplicate with cycling parameters similar to those described above for the amplification efficiency analysis.

### Stability analysis

To validate and compare the suitability of the candidate reference genes for use in normalization, we evaluated their expression stability in response to drought along the day under the experimental conditions described above. For this purpose, Cycle threshold values (Ct) were transformed into non-normalized relative quantities (Q; linear scale). Here, Q = EΔCt, where E is the amplification efficiency, and ΔCt is the lowest Ct from the data set minus the sample Ct. The non-normalized relative quantities were analyzed using NormFinder [33] and geNorm [34] software to assess the expression stability of the reference genes.

### Normalization of target gene expression

The relative expression level of the drought-responsive gene *GmDREB5*-like [30] was measured in leaf samples from BR16 plants subjected to moderate drought, sampled over a 24 h timecourse, under the experimental conditions described above. For each time point (ZT0, ZT4, ZT8, ZT12, ZT16 and ZT20), three biological replicates, with three technical replicates each, were analyzed. Target expression was normalized using a combination of 2 reference genes with high (*FYVE* and *GOL84*), intermediate (*ELF1-β* and *β -actin*) and low (*DNAJ* and *NCL1*) expression stabilities. Plants grown under normal water conditions (control plants) were used to calibrate relative expression.

The gene expression analysis was performed using the Rest2009 software package [35], which allows the input of different amplification efficiencies for the reference and target genes and provides the statistical significance of expression levels through randomization (Pair Wise Fixed Reallocation Randomisation Test©), with 10,000 interactions and bootstrapping of the data. At the randomization tests, the observed values were repeatedly and randomly reallocated to the two groups and the apparent effect (expression ratio in our case) was noted each time. The proportion of these effects which are as great as that actually observed in the experiment gave us the P-value of the test. In the applied Pair Wise Fixed Reallocation Randomisation Test©, for each sample, the CP values for reference and target genes were jointly reallocated to control and sample groups (= pairwise fixed reallocation), and the expression ratios was calculated on the basis of the mean values. Hypothesis testing was conducted to determine whether the differences between the control and treatment conditions were significant [35].

## Results and Discussion

### Screening of candidate reference genes

To date, most of the studies on reference genes have focused on validating a subset of commonly used reference genes for specific contexts [15,16,18]. Although these studies have their merits, they attempt to identify the best candidates from a small set of genes. A recent analysis demonstrated that reference genes are preferably selected by adopting a complete genome strategy, rather than from a handful of commonly used reference genes [26]. In this context, we searched reference genes showing high expression stability in 59 microarray libraries from soybean subjected to drought stress, heat and different photoperiods [26].

**Fig 1:**
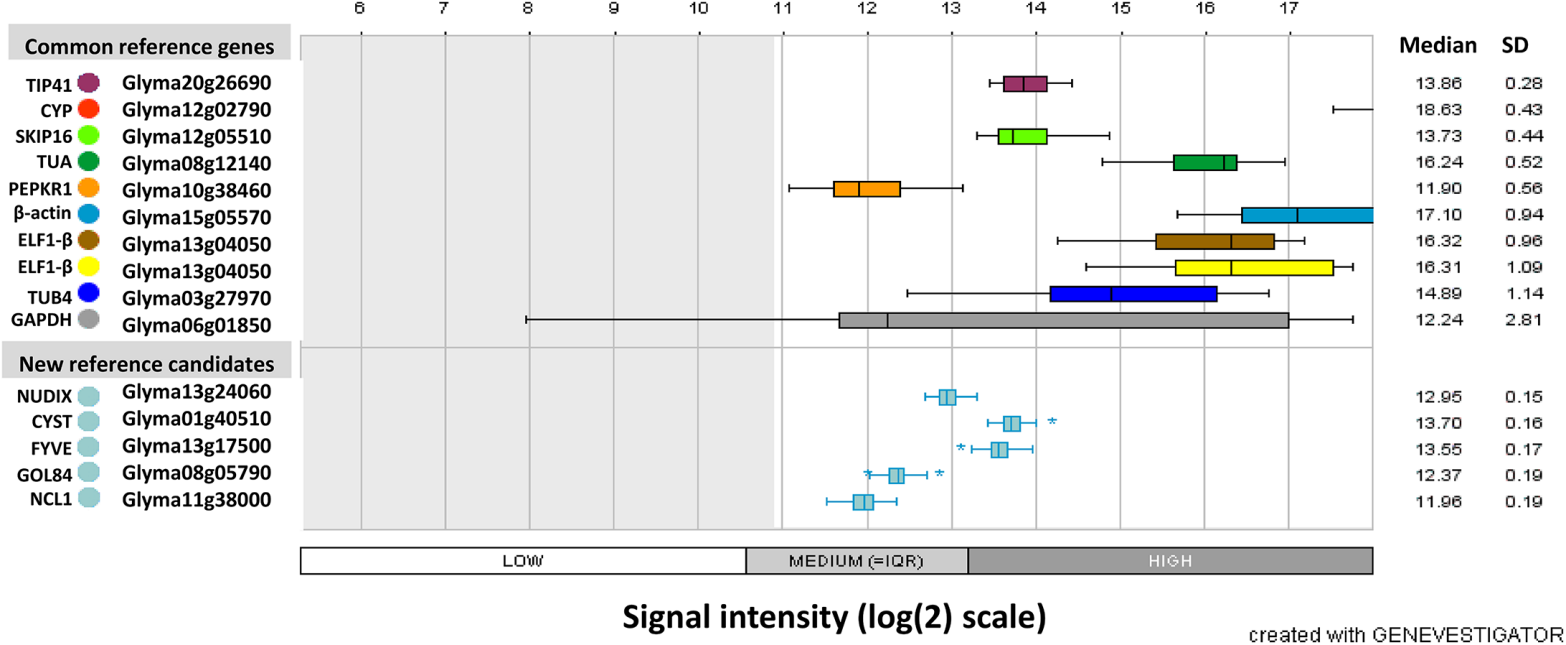
Expression of commonly used reference genes and new candidates from microarray databases. The *in silico* analyses were performed with Genevestigator software, using data from soybean subjected to drought stress, heat and different photoperiods[27]. The box plots represent the variation in the signal intensity (log_2_ scale). Interquartile range (IQR) values are shown for each gene across the dataset, represented by the middle boxes, which encompass the middle 50% of scores for the group. The upper and lower whiskers represent scores outside the middle 50%. Outliers are plotted separately as asterisks on the chart.

This tool allowed us to perform *in silico* identification of genes showing high expression stability in 59 microarray libraries from soybean subjected to drought, heat and distinct light periods. The candidate reference genes obtained in this analysis were pre-validated by checking their expression across all microarrays available on the Genevestigator platform (3458 arrays) (data not shown). The expression profiles of the five most stable new reference genes in response to drought and to diurnal oscillations was compared with the expression of the commonly used soybean reference genes (Table 1), as shown in Fig 1.

**Fig 2:**
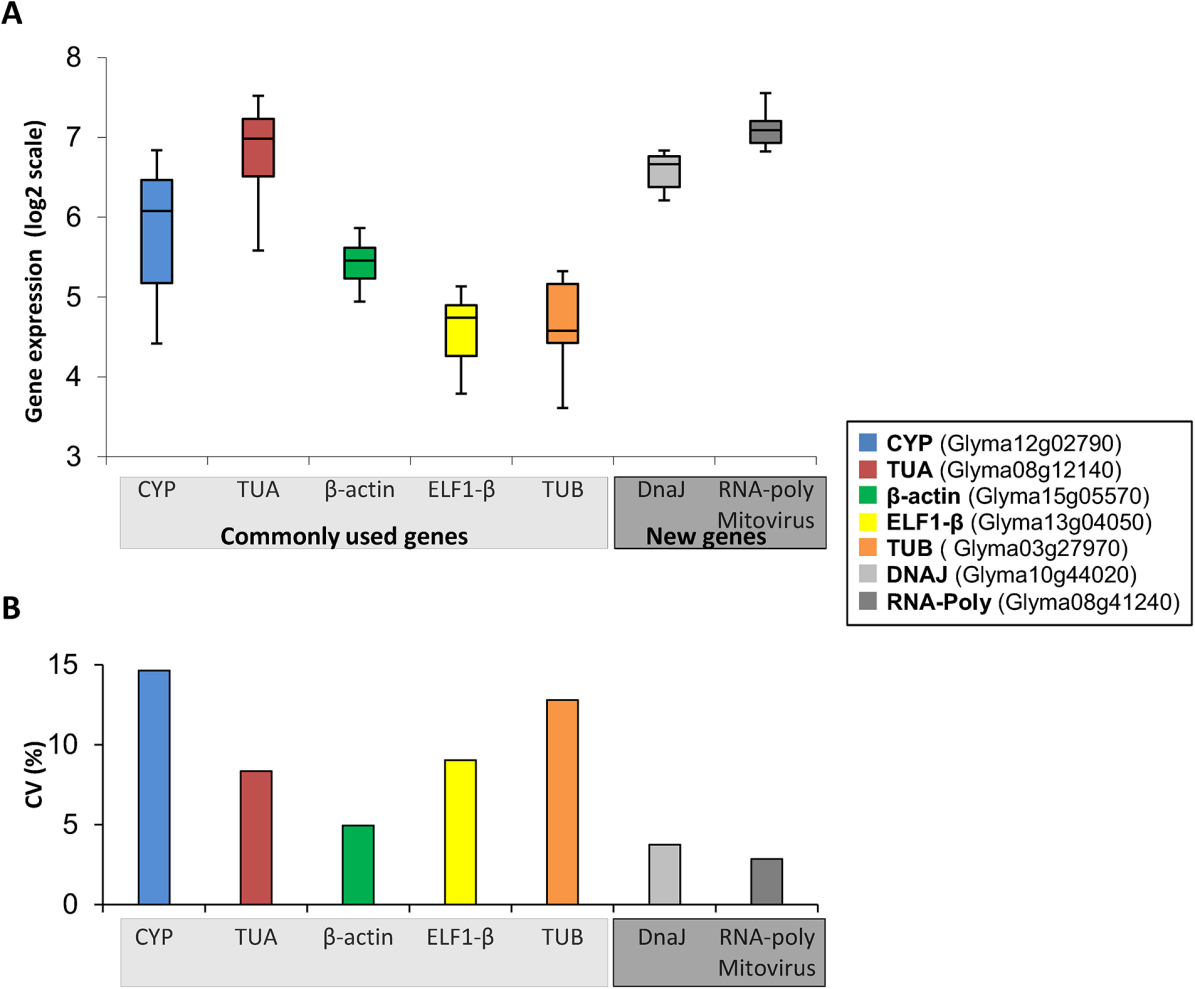
Variation in the expression of commonly used reference genes and new candidates from the RNA-Seq database. The *in silico* gene expression analyses were performed using data from soybean grown under drought stress across a 24 h timecourse. (A) The box plots represent the variation in the signal intensity (log2 scale); the middle boxes represent the middle 50% of scores for the group; the upper and lower whiskers represent scores outside the middle 50%. (B) Coefficient of variation (CV%).

Then, in a second approach, we selected genes that exhibited minimal expression variance across 36 cDNA libraries synthesized from drought-stressed soybean plants sampled over a 24 h timecourse [10,36]. The expression profiles of the two most stable new reference genes and the commonly used references are shown in Fig 2.

In summary, the data mining approaches using microarray and RNA-seq databases allowed us to select a new set of candidate reference genes for validation via RT-qPCR, composed of seven soybean genes: Glyma13g24060, Glyma01g40510, Glyma13g17500, Glyma08g05790, Glyma11g38000, Glyma08g41240 and Glyma10g44020.

The majority of the selected candidate genes are related to the plant’s primary metabolism. For example, Glyma01g40510 encodes a cysteine desulfurase (CYST) similar to nitrogen fixation S (NIFS)-like 1 from *Arabidopsis*; Glyma08g05790 encodes a protein that participates in Golgi vesicles transport (Golgin-84) [37,38]; Glyma11g38000 produces an RNA (cytosine-5)-methyltransferase (NCL1) involved in epigenetic modifications of tRNA [39,40]; and Glyma13g17500 produces an FYVE domain protein, present in kinases and lipases in *Arabidopsis*, that recognizes phosphoinositide signals [41].

The differential expression of some of the selected candidate genes has been reported during biotic and abiotic stress responses. The gene Glyma13g24060, for example, encodes a protein similar to a NUDIX hydrolase protein from *Arabidopsis*. The NUDIX hydrolase family is widespread, from eukaryotes to *Archaea*, and consists of pyrophosphohydrolases that act upon substrates with a general nucleoside diphosphate structure, including (deoxy)ribonucleoside diphosphates and triphosphates, nucleotide sugars, coenzymes and RNA caps [42,43]. Members of the NUDIX family have been reported to be induced by salt, drought, heat, and cold in *Chrysanthemum lavandulifolium* [44]. Similarly, Glyma10g44020 encodes a protein from the DnaJ/Hsp40 cysteine-rich domain superfamily, which is described as being involved in diverse cellular processes (protein folding, translocation, and degradation) [45], including biotic and abiotic stress responses [46–49]. However, analysis of the soybean *NUDIX* (Fig 1) and *DNAJ* (Fig 2) genes in microarray and RNA-Seq databases showed high expression stability in response to drought and diurnal oscillations, suggesting that these soybean genes could be reliable candidate reference genes for drought studies.

Furthermore, we identified a gene (Glyma08g41240) that encodes an RNA-dependent RNA polymerase from a mitovirus (Fig 2). A recent study on the soybean mitochondrial genome revealed the presence of a 0.5 kb insertion (at *rps10* intron) that is 57.4% identical to a mitovirus RNA polymerase gene, which might have been horizontally transferred during recent evolution. Although the effect of this insertion remains unknown, analysis of the insert’s position suggests that it might affect the function of the mitochondrial *rps10* gene, which encodes the ribosomal protein S10 [50].

The preliminary analysis of this set of genes (Figs 1 and 2) revealed that in the majority of cases, the candidate reference genes presented less variation than the commonly used reference genes selected from the literature [15,19]. Additionally, *in silico* pre-validation of the *NUDIX*, *CYST*, *FYVE*, *Golgin-84*, and *NCL1* genes across 3,458 microarrays using GeneVestigator platform revealed that these genes are unresponsive to a wide variety of conditions, including abiotic stresses, such as heat, salinity, and cold, and show little variation between developmental stage and genotypes, being responsive only to infections by *Phytophthora sojae*, *Phakopsora pachyrhizi* and *Bradyrhizobium japonicum* (data not shown).

### Stability analysis

To experimentally validate this new set of candidate reference genes, we examined their individual properties and compared the stability of their expression with the commonly used reference genes *ELF1-β* and *β-actin* (Table 1) using RT-qPCR assays. In previous studies, *ELF1-β* and *β-actin* showed high expression stability across different levels of drought [15,28,30]. However, no study conducted to date has investigated the expression stability of these genes during the diurnal cycle. Validation was carried out on soybean leaves from plants subjected to a moderate water deficit (30% of gravimetric humidity (GH)), sampled across a 24 h timecourse, with 4 h intervals, from 8:00 a.m. to 4:00 a.m., corresponding to Zeitgeber Time (ZT) 0 to ZT20. The expression level of each reference gene was evaluated separately in drought-treated and control plants at all sampling times (ZT0, ZT4, ZT8, ZT12, ZT16 and ZT20).

**Fig 3:**
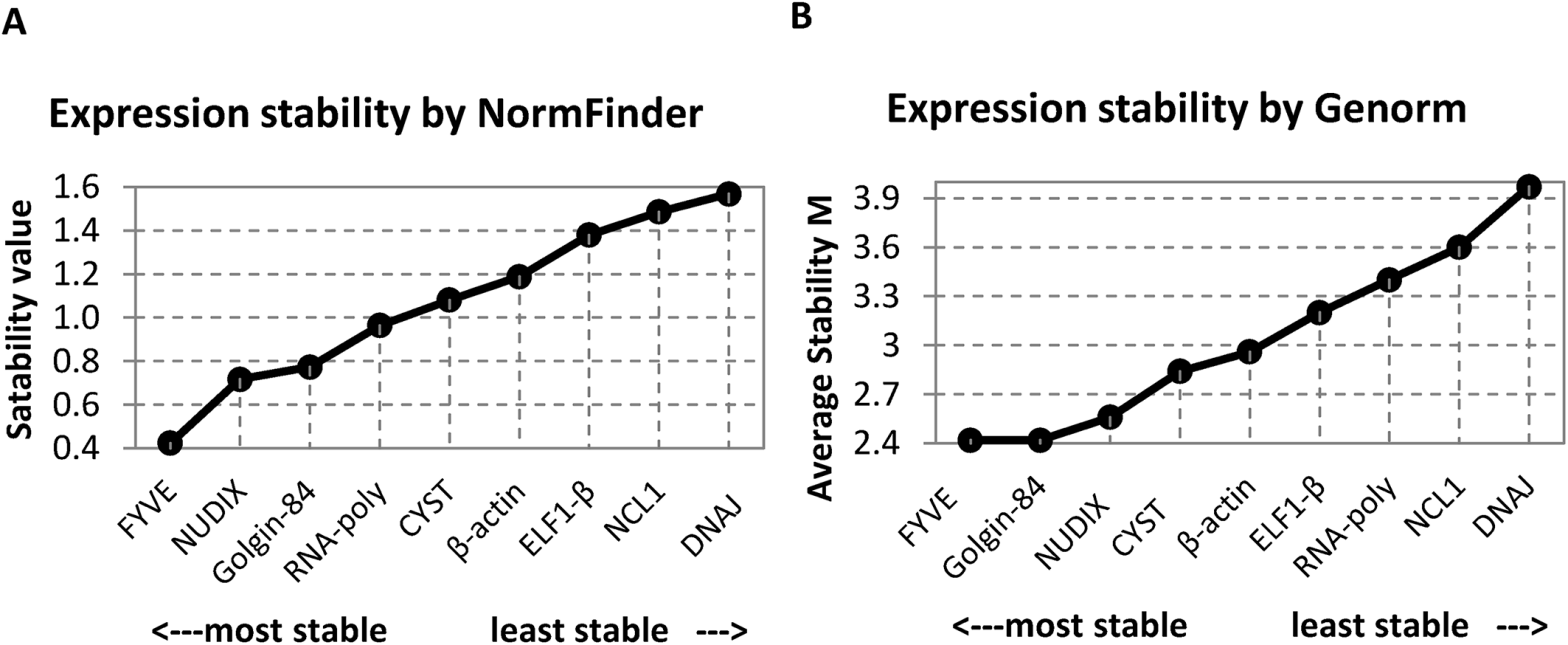
Stability analysis of candidate reference genes. Gene expression stability was measured in leaf tissues of soybean subjected to drought stress and control conditions at different times of day (ZT0, ZT4, ZT8, ZT12, ZT16 and ZT20). The analysis was performed with NormFinder and geNorm softwares. Genes were ranked according to their stability values and *M values* from **(A)** NormFinder and **(B)** geNorm, respectively. The genes were plotted on the x-axis from the most to the least stable.

To assess the stability values for the reference genes, we performed analysis using the NormFinder [33] and geNorm softwares [34]. The NormFinder software employs a variance estimation approach to rank genes according to combined inter- and intragroup expression variation across a given set of experimental conditions, which were drought stress and diurnal oscillations in this case. The NormFinder [33] is known to perform in a more robust manner and with less sensitivity toward the co-regulation of candidate genes compared with other software. On the other hand, the NormFinder software may be less robust with small sample sizes compared to the geNorm algorithm [33]. Thus, we performed additional stability analysis using geNorm software [34] to compare the performance of the candidate genes using stability analysis tools with different algorithms. In general, the results of geNorm analysis were similar to those from NormFinder, with slight differences regarding ranking positions (Fig 3A and 3B). This good consistence between both outcomes strengthens the robustness of the results obtained. Small differences in rank position among the two software are expected because the statistical algorithms used are distinct: the geNorm detects the two reference genes whose expression ratios show the least variation from those of the other tested genes [34], whereas the NormFinder takes intra- and intergroup variation into account for calculations [33].

According these stability analysis, many of the newer reference genes indeed exhibited greater expression stability than the conventionally used reference genes (*ELF1-β* and *β -actin*) (Fig 3A and 3B). The *FYVE*, *NUDIX* and *Golgin-84* genes were the most stable, suggesting that they are the most suitable for normalizing expression data from combined studies addressing drought treatment and diurnal oscillation (Fig 3A and 3B).

**Fig 4:**
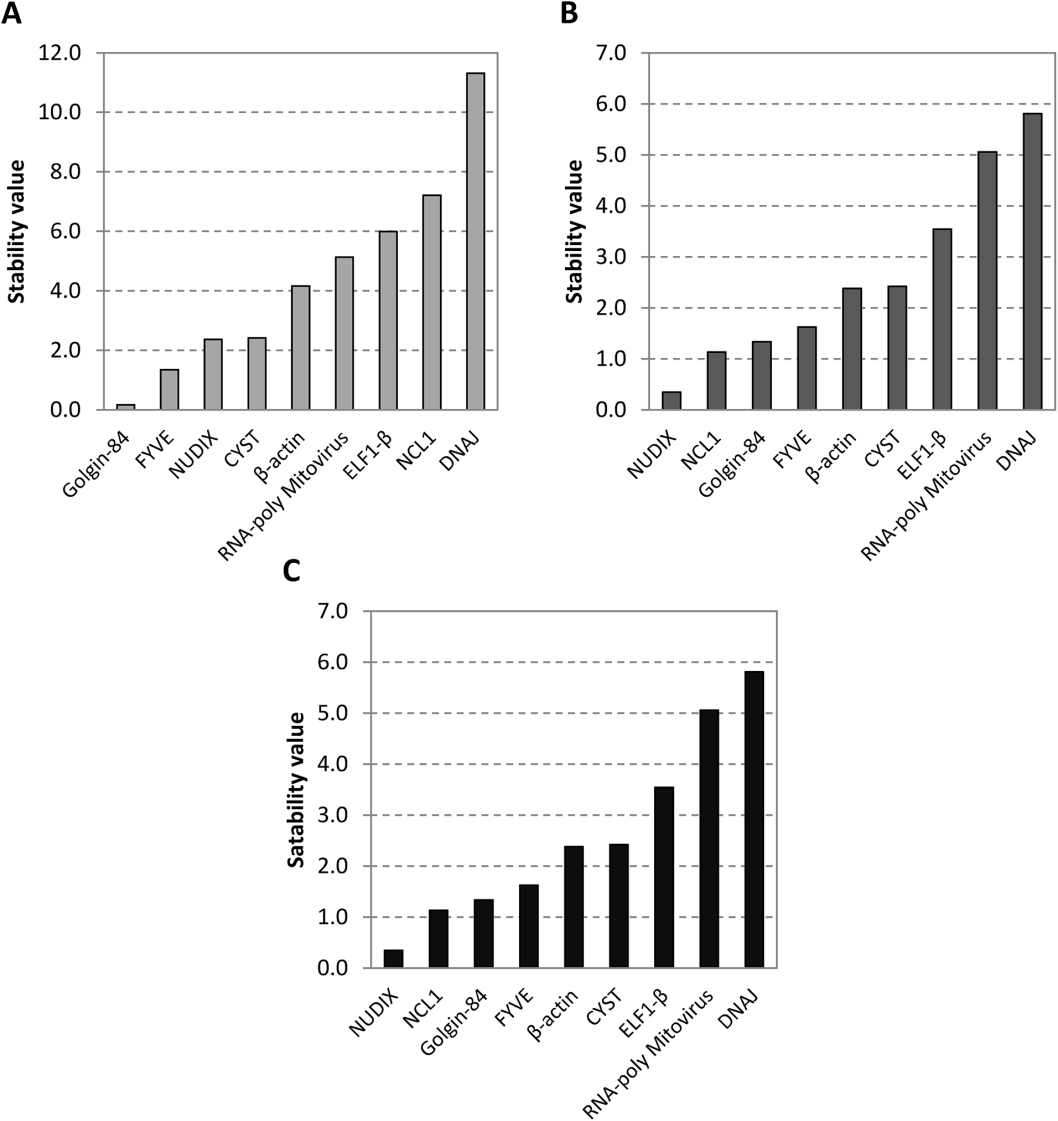
Intra- and intergroup variation of gene expression. The intragroup variation within the **(A)** control (non-stressed plants) and **(B)** stressed (plants under drought stress) and **(C)** the intergroup variation are presented.

Although most of the candidate reference genes performed well in response to the applied experimental conditions, the *NCL1* and *DNAJ* genes showed lower stability (Fig 3A and 3B). The *NCL1* gene produces an RNA (cytosine-5)-methyltransferase involved in epigenetic modifications of tRNA [39,40]. The analysis of intragroup variation performed on NormFinder for *NCL1* revealed that under control conditions this was one of the least stable genes (Fig 4A), whereas in stressed plants it was among the most stable (Fig 4B), indicating that *NCL1* is not a suitable reference gene for studies on gene expression oscillation during the day in plants under control conditions. Previous studies show that the oscillation of genes that cycle daily in normal conditions may be altered due the imposition of abiotic stresses, like cold and drought stress. The mRNA levels of some chestnut genes, like *TOC1* and *LHY* cycle daily in seedlings and adult plants, as expected. However, during chilling stress (4°C), mRNA levels of these genes were higher and did not oscillate [51]. Similar events were observed in soybean plants subjected to severe drought stress, where a general reduction in the amplitude of the daily oscillation was observed for most clock genes, including the *GmPRR3-like*, *GmPRR7-like*, *GmPRR9- like*, *GmGI-like*, *GmZTL-like*, and *GmCHE-like* genes [10].

Furthermore, these results illustrate that gene expression stability during the day may vary in response to stressful conditions, like changes in the plant’s water status (e.g. normal hydration versus water deficit conditions).

In contrast, the expression of the *DNAJ* gene was unstable in both control and stressed conditions (Fig 4). Genes from the DNAJ family have been reported to be drought responsive in many species, and its lack of stability may therefore be explained in part by its possible involvement in drought responses in soybean, as studies have reported that drought-responsive genes oscillate in response to diurnal oscillations [10,12,13,30,52]. Additionally, the intragroup analysis shows that *Golgin-84* and *FYVE* (Fig 4A) are the most reliable reference genes for gene expression normalization when studying diurnal oscillations in plants under normal water conditions, whereas *NUDIX* and *NCL1* (Fig 4B) are the most strongly indicated for use in studies under drought conditions. Furthermore, the intergroup variation analysis allowed us to identify the genes *NUDIX* and *DNAJ* as the most and least stable genes, respectively, for data normalization in studies comparing gene expression under control and drought stress, without considering the time of day (Fig 4C).

### Normalizing the expression of a target gene

**Fig 5:**
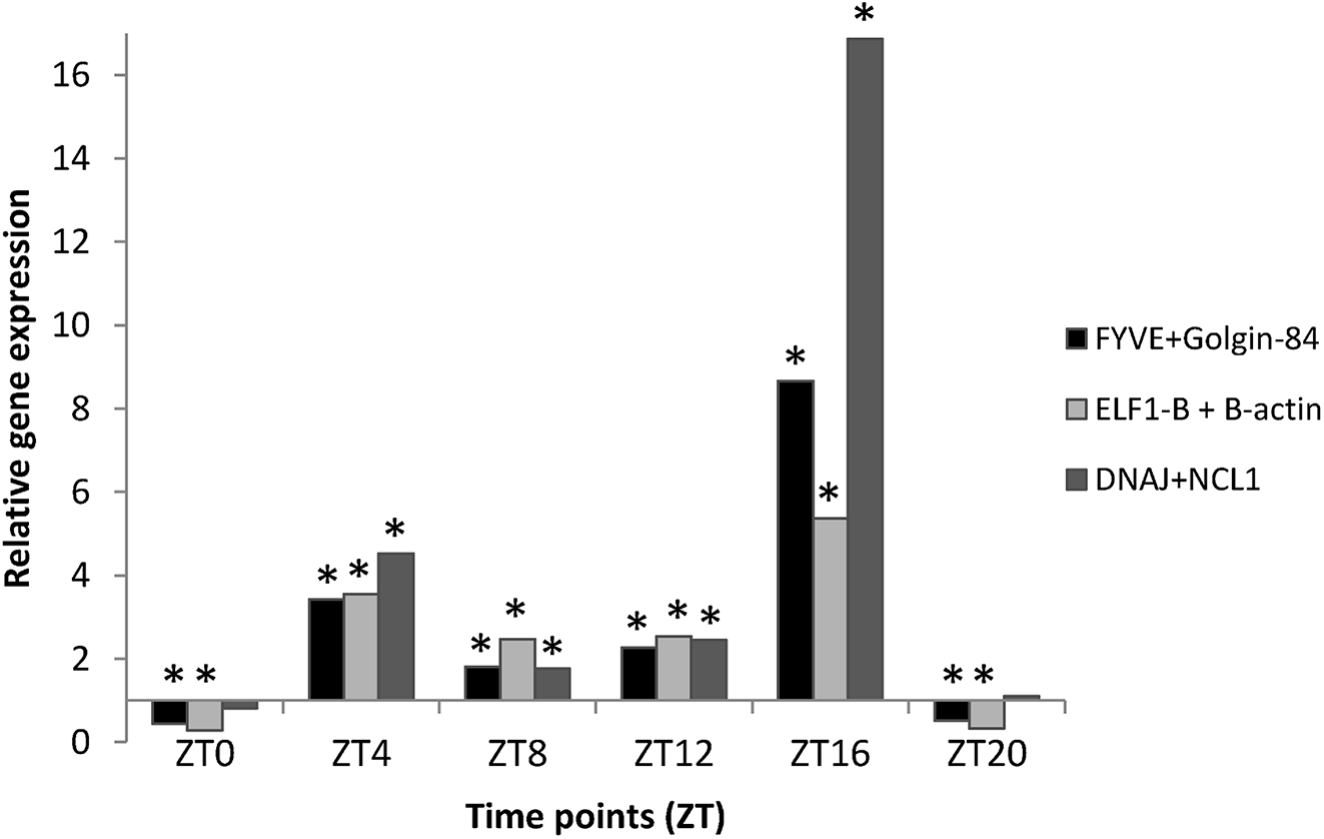
Normalization of the target gene *GmDREB5*-like. Gene expression was measured in leaf tissues of soybean subjected to drought stress at different times of day (ZT0, ZT4, ZT8, ZT12, ZT16 and ZT20). The raw data were normalized using a combination of 2 candidate reference genes with high (*FYVE* and *GOL84*), intermediate (*ELF1-β* and *β -actin*) and low (*DNAJ* and *NCL1*) expression stabilities. The relative expression of *GmDREB5*-like under drought stress was determined after comparison with the control sample (non-stressed plants). Asterisks indicate statistically significant changes in gene expression between drought stressed and non-stressed plants. The analyses were performed using REST 2009 software.

A previous study showed that the normalization of soybean genes under circadian regulation using unstable reference genes may lead to erroneous data interpretation [19]. To demonstrate the effect of data normalization using reference genes with different stability values in response to drought stress during the day, we evaluated the relative expression of the drought-responsive gene *GmDREB5*-like [30] using a combination of 2 reference genes with high (*FYVE and GOL84*), intermediate (*ELF1-β* and *β -actin*) and low (*DNAJ and NCL1*) expression stabilities (Fig 5). The *GmDREB5* gene expression was previously investigated in response to drought in short-term stress conditions [30], and its expression was normalized by *ELF1-β* and *β –actin*. As expected, our results show that the expression of *GmDREB5*-like in response to drought oscillates during the day, as previously described for this gene [30] and for other genes of the DREB subfamily [10,53–58] (Fig 5).

However, our results demonstrated that choosing reference genes with diverse stability of expression can lead to differences on gene expression data interpretation when evaluating combined studies on water deficit and diurnal oscillations. As shown in Fig 5, at ZT16 the normalization of *GmDREB5*-like gene using the least stable genes (*DNAJ and NCL1*) resulted in higher expression levels than observed for normalization using genes with high (FYVE and GOL84) and intermediate expression stability (ELF1-β and β –actin). Additionally, slight changes in gene expression, such as the down-regulation of the target gene at ZT0 and ZT20, were detected only by normalization using genes with high (*FYVE* and *GOL84*) and intermediate expression stability (*ELF1-β* and *β –actin*) (Fig 5A). These results emphasize the importance of selecting reference genes with stable expression for accurate gene expression analysis on drought responses and diurnal oscillations.

## Conclusions

Here, by analyzing experiments involving both drought and diurnal oscillations, we demonstrated the importance of selecting reference genes under the specific studied conditions. From a transcriptome-wide background, we selected a new set of candidate reference genes for the normalization of data obtained in studies on drought and diurnal oscillations.

The experimental validation of this new set of candidate reference genes revealed that *FYVE*, *NUDIX* and *Golgin-84* were the most stably expressed genes in soybean plants under control and drought conditions along the day, and are therefore considered the best reference genes for the studied conditions. Our results highlight that the selection of reference genes is crucial for the proper quantification of relative expression data obtained under these specific experimental conditions.
